# Spectroelectrochemical Assessment of Polypyrrole Formation Dynamics on Silane-Modified Surfaces and Application to pH Sensing

**DOI:** 10.1021/acsmeasuresciau.6c00155

**Published:** 2026-07-03

**Authors:** Deivis Plausinaitis, Ernestas Brazys, Gintautas Bagdziunas, Almira Ramanaviciene, Vilma Ratautaite, Arunas Ramanavicius

**Affiliations:** † Department of Physical Chemistry, Institute of Chemistry, Faculty of Chemistry and Geosciences, Vilnius University (VU), Naugarduko str. 24, Vilnius LT-03225, Lithuania; ‡ Department of Nanotechnology, State Research Institute Center for Physical Sciences and Technology (FTMC), Sauletekio Ave. 3, Vilnius LT-10257, Lithuania; § Group of Supramolecular Analysis and Bioelectronics at Institute of Biochemistry, Life Sciences Centre, Vilnius University, Sauletekio av. 7, LT-10257 Vilnius, Lithuania; ∥ Nanotechnas−Centre of Nanotechnology and Materials Science, Institute of Chemistry, Faculty of Chemistry and Geosciences, Vilnius University (VU), Naugarduko str. 24, Vilnius LT-03225, Lithuania

**Keywords:** conducting polymers, polypyrrole (PPy), electrochromic polymers, indium tin oxide (ITO) electrodes, surface silanization

## Abstract

Interfacial processes during the electrodeposition of conducting polymers critically dictate layer growth, adhesion, and charge transfer behavior. In this work, polypyrrole (PPy) layers were electrodeposited onto indium tin oxide (ITO) electrodes modified with triethoxymethylsilane (TEMS) or trimethoxyvinylsilane (TMVS) using a double potential step (DPS) method. The deposition was investigated using *in situ* light absorption spectroscopy (λ = 530 and 750 nm) coupled with chronoamperometry. The results showed that conducting polymer formation involves an initial growth stage, an accelerated polymerization, and a transition to stable growth. The oxidation-to-reduction charge ratio remained >1, indicating net layer growth during successive pulses. The choice of the silane precursor modulated the polymerization kinetics and the relative abundance of polaronic and bipolaronic species. The PPy layers exhibited pH-dependent changes in optical absorption, where the TMVS-modified electrode showed a broader sensing range. These findings demonstrate that *in situ* spectroelectrochemical monitoring is effective for elucidating polymer growth and that silane-modified ITO/PPy structures hold potential for optical sensing applications.

## Graphical abstract

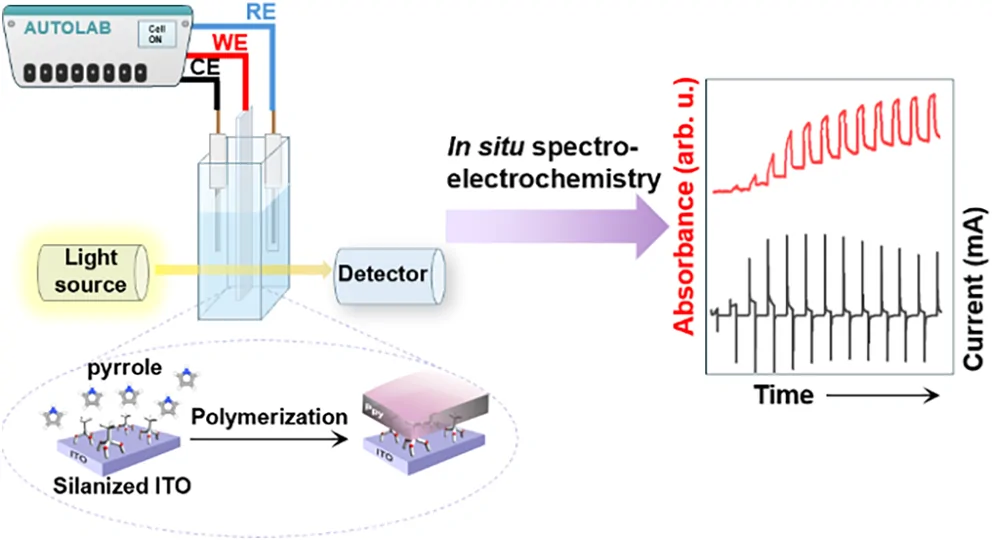

## Introduction

Conducting polymers have been widely investigated for applications in supercapacitors,^1,2^ fuel cells,^3,4^ electrochromic devices,^5,6^ and sensor systems.^7,8^ Among them, polypyrrole (PPy) is one of the most extensively studied conducting polymers. Conventionally, PPy can be synthesized by two main approaches: (i) oxidative chemical polymerization using oxidants such as FeCl_3_,^9,10^ ammonium persulfate (APS),^11^ or hydrogen peroxide,^12–15^ as well as less common enzymatic^16,17^ techniques, and (ii) electrochemical polymerization employing electrochemical techniques such as cyclic voltammetry,^18,19^ potentiostatic,^20^ and pulsed methods.^7^ Electrochemical polymerization enables controlled deposition of the polymer on electrically conductive regions of the electrode surface, whereas chemical methods typically produce a bulk material. For some applications, the self-assembly and diffusion of monomers and other molecules present in the polymerization solution (e.g., template molecules for molecularly imprinted polymer preparation) might have an important role and can be influenced by the application of the double potential step (DPS) method.^21^ A sequence of short, low- and high-potential pulses is applied in the DPS method. During the high-potential pulse, monomer oxidation and polymer formation occur, while the low-potential pulse allows diffusion and replenishment of the monomer, dopant ions, and other electroactive species near the electrode surface.^22,23^ In addition to the processes occurring in solution, the properties of the electrode surface also influence the formation and characteristics of the resulting polymer film. The stability and adhesion of the PPy layers depend on the electrode material and the nature of polymer–electrode interactions.^24^ Therefore, electrode surface pretreatment is a critical step prior to electrochemical polymerization.^25–27^ Moreover, processes occurring at the electrode surface during deposition affect how the polymer forms. They influence charge transfer, layer formation behavior, and the structure of the resulting polymer layer. As a result, electrode surface modification is an effective approach to tune the properties and performance of electrochemically deposited conducting polymers.^28^ Also, electrografted monolayers of vertically oriented polyaniline oligomers can be formed on silane-modified ITO surfaces, where such a configuration enhances layer stability and provides excellent pH-sensing properties.^29^

PPy layers have been widely investigated for electrochromic applications, such as smart windows^30,31^ and optical sensing devices,^19^ due to their ability to undergo reversible changes in optical properties under an applied potential. These properties are associated with electrochromism, defined as changes in the optical properties of materials induced by electrochemical oxidation and reduction.^32,33^ The electrochemical behavior of PPy is commonly interpreted using the polaron–bipolaron model, in which oxidation of the neutral polymer forms polaronic species, and at higher doping levels, bipolaronic species become dominant. Importantly, the studies indicate that these charge carriers can coexist over a range of intermediate oxidation states rather than forming well-defined, distinct states.^34–36^ The optical response of PPy is therefore better described as a set of broad, oxidation-dependent absorption regions arising from multiple electronic transitions, rather than as a few well-defined peaks.^37–40^ In the reduced state, PPy is characterized by a π–π* transition in the near-UV region, typically around 300–400 nm, the exact position of which depends on the polymer structure and layer morphology.^37,39,40^ Upon oxidation, the π–π* band decreases in intensity, while new absorption bands at longer wavelengths arise due to the formation of polaronic and bipolaronic energy levels within the band gap.^35,36,38^ A visible absorption band associated with the polaron formation is commonly observed in the range of 400–600 nm. However, it is typically broad and not sharply defined, often appearing within a broader spectral region.^37,38^ With increasing oxidation (higher doping levels), the optical response shifts toward a broad absorption in the red-to-near-infrared region beyond 700 nm, often reaching the 800–900 nm range, which is characteristic of highly doped PPy and is commonly attributed to bipolaronic or delocalized charge carrier states.^37,39,40^ Overall, spectroelectrochemical studies suggest that PPy is better described in terms of continuous spectral changes and overlapping charge carrier bands, rather than absorption maxima associated with specific redox states.^37–40^

The electrochromic behavior described above can be investigated using spectroelectrochemical techniques, in which changes in optical response are monitored under electrochemical control. In this context, optically transparent electrodes, particularly ITO- and fluorine-doped tin oxide (FTO)-coated substrates, are commonly employed. Most studies report measurements of fully formed PPy layers in monomer-free electrolytes, in which reversible redox switching, typically achieved by cyclic voltammetry or potential step methods, enables evaluation of their optical response.^37,41–46^ Spectroelectrochemical measurements can also be performed during electrochemical polymerization of PPy, enabling monitoring of optical changes as the layer forms. Although it is less commonly reported, this approach can provide direct insights into changes in the electronic structure and electrochromic properties of the growing layer, as well as into the processes involved in layer formation. In the study of Kim et al.,^47^ *in situ* UV–Vis spectroelectrochemical measurements carried out during the electropolymerization of pyrrole in acetonitrile containing 0.1 M tetrabutylammonium perchlorate showed the rapid formation of oligomeric species, with absorption bands shifting from around 340 nm toward 540 nm, characteristic of the polaronic state, and then shifting to longer wavelengths in the 600–750 nm region and above, associated with bipolaronic species. Kinetic analysis showed fractional reaction orders of approximately 1/3–1/2 for the monomer and about 1/3 for the deposited polymer, indicating that both contribute to growth, with early-stage oligomer formation as the rate-limiting step. These findings demonstrate that electrochromic behavior can be used to monitor the polymerization process itself and that further *in situ* spectroelectrochemical studies could provide valuable insights into the fundamental mechanisms of layer formation, improving control over material properties for practical applications.

This study investigates how interfacial surface modification and polymerization conditions influence the *in situ* electrochromic behavior of polypyrrole during electrochemical layer formation. PPy layers were deposited on ITO-coated electrodes modified with two different silanes by using a DPS method, enabling controlled growth under alternating oxidation and reduction pulses. Simultaneous electrochemical and optical measurements were used to monitor changes in absorbance at characteristic wavelengths associated with polaronic and bipolaronic states throughout the layer formation. By correlating the charge passed during polymerization with changes in optical absorbance, the relationship among charge transfer, polaronic, and bipolaronic states and layer growth was established. The resulting layers were further evaluated for optical pH sensing.

## Results and Discussion

The silanes bearing methyl (TEMS) and vinyl (TMVS) functional groups were deposited on the ITO surface. The electrochemical formation of PPy was carried out on the ITO_(TEMS)_ and ITO_(TMVS)_ electrodes. Two different silanes, in which TEMS contains a methyl group and three ethoxy groups and TMVS contains a vinyl group and three methoxy groups attached to the central silicon atom, were compared. Both silanes are electrochemically inactive, do not participate in the PPy reaction, and are used to tune the ITO surface’s hydrophobic properties. The resulting PPy layer exhibits improved mechanical stability on the electrode surface while remaining easily removable from the ITO electrode.^19^ Our previous study compared the polymerization of PPy on unmodified ITO and silane-modified ITO. The results of this study demonstrated that PPy polymerization on bare and modified electrodes proceeds markedly differently.^19^

These differences, determined by the polymerization procedure, were identified by cyclic voltammetry (CV) through the analysis of oxidation- and reduction-related features in the cyclic voltammograms. Here, the PPy layer was formed by applying a DPS-based electrochemical deposition method. A comparison of the polymerization using the DPS on the ITO_(TEMS)_ and ITO_(TMVS)_ electrodes is presented in Figure 1. The differences in the polymerization process were further evaluated by *in situ* monitoring of the optical absorbance at 530 and 750 nm.

**Figure 1:**
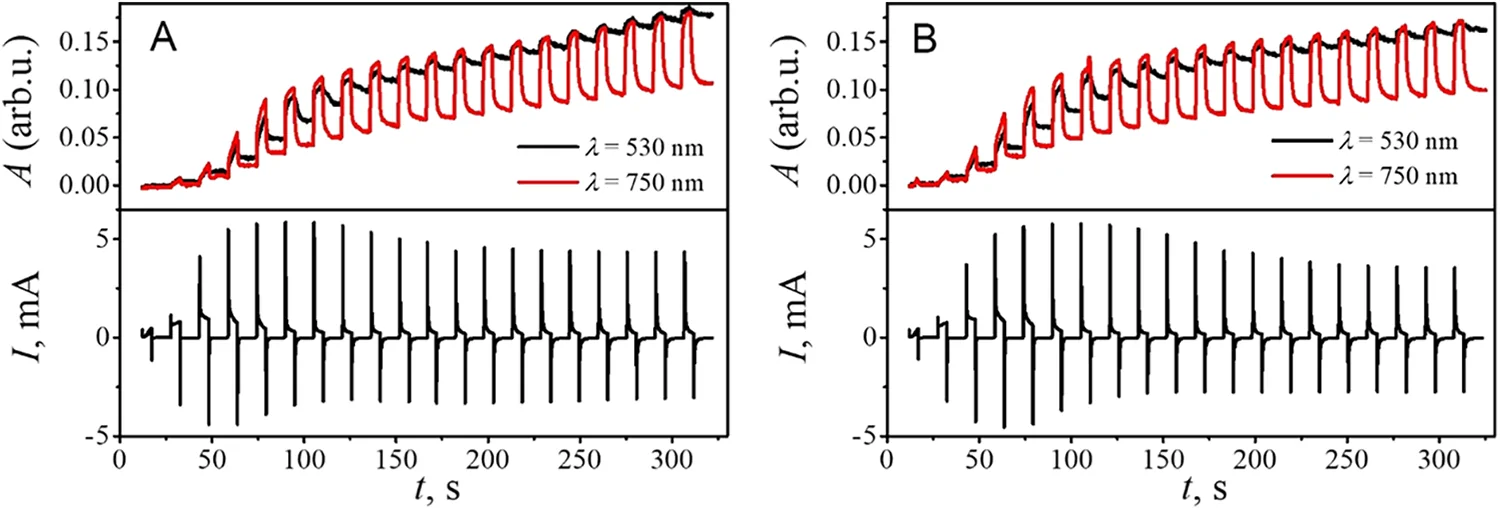
Changes in current *I* and absorbance *A* during the electrochemical formation of PPy on: (A) ITO_(TEMS)_ and (B) ITO_(TMVS)_ electrodes by applying a DPS protocol of +1.0 V for 5 s, followed by 0 V vs Ag/AgCl_(3 M KCl)_ for 10 s, for a total of 20 pulses. The changes in absorbance were followed at 530 and 750 nm, respectively.

The optical properties of the electrodeposited PPy layers are most commonly investigated after the polymerization, typically by transferring the polymer-coated electrode into a monomer-free electrolyte solution and recording visible light absorption spectra under controlled electrochemical conditions, as discussed in the Introduction section. Here, however, the evolution of the optical properties was monitored *in situ* during the electrodeposition process under polymerization conditions. As described above, electrochemical polymerization was carried out using the DPS method, in which the potential of the ITO_(TEMS)_ or ITO_(TMVS)_ electrode was alternated between +1.0 V for 5 s and 0.0 V vs Ag/AgCl_(3 M KCl)_ for 10 s for a total of 20 pulses at each potential. The oxidation potential pulse was selected based on cyclic voltammetry (CV) studies in our previous work.^19^ Pyrrole oxidation has been reported to begin at potentials as low as +0.65 V vs Ag/AgCl, depending on the experimental conditions.^48^ +1.0 V was selected to ensure efficient oxidation of pyrrole and the formation of PPy. The use of DPS enables pyrrole oxidation during the high-potential pulse, whereas the low-potential pulse may facilitate diffusion and replenishment of electroactive species near the electrode surface.^23^ In contrast to CV, where polymer growth occurs under continuously changing potentials, DPS allows polymerization to proceed at a constant anodic potential, which may contribute to more controlled film growth. Figure 1A,B shows changes in electrochromic behavior during the PPy layer formation under the selected DPS conditions, monitored *in situ* through visible absorbance at 530 and 750 nm, respectively. These wavelengths were selected based on our previous study,^19^ in which absorption bands with maxima at 530 and 750 nm were observed for the fully formed electrodeposited PPy layers. While previous work examined fully formed PPy layers after deposition, these results reveal the evolution of the optical and electrochromic properties during PPy layer formation.

The identified current values are shown in Figure 2A, and those during a +1.0 V potential step are shown in Figure 2B,C. The current recorded at the start of the pulse is associated with rapid electrochemical processes such as double-layer charging. Therefore, it is denoted as *I*_c_ (capacitive current). Meanwhile, the current measured at the end of the pulse is related to slower processes, such as diffusion of substances, and is denoted as *I*_D_. Trends in both cases were similar. During the electrochemical polymerization, the initial current *I*_C_ of the electrode reaction increased from the first to the seventh potential step. In subsequent pulses, current *I*_C_ decreased and stabilized at approximately 4.3 mA for the PPy-modified ITO_(TEMS)_ electrodes and 3.5 mA for the ITO_(TMVS)_ electrodes. At the end of the electrode reaction, the current *I*_D_ value increased only from the first to the third potential step. During subsequent potential steps, current *I*_D_ decreased and stabilized at 0.22 mA for the PPy-modified ITO_(TEMS)_ electrodes and 0.19 mA for the PPy-modified ITO_(TMVS)_ electrodes. Regarding similar trends, the differences between Figure 2A,B are not apparent until the signals of each pulse are analyzed in detail. The differences increase with the number of applied potential pulses, as shown in the following figures.

**Figure 2:**
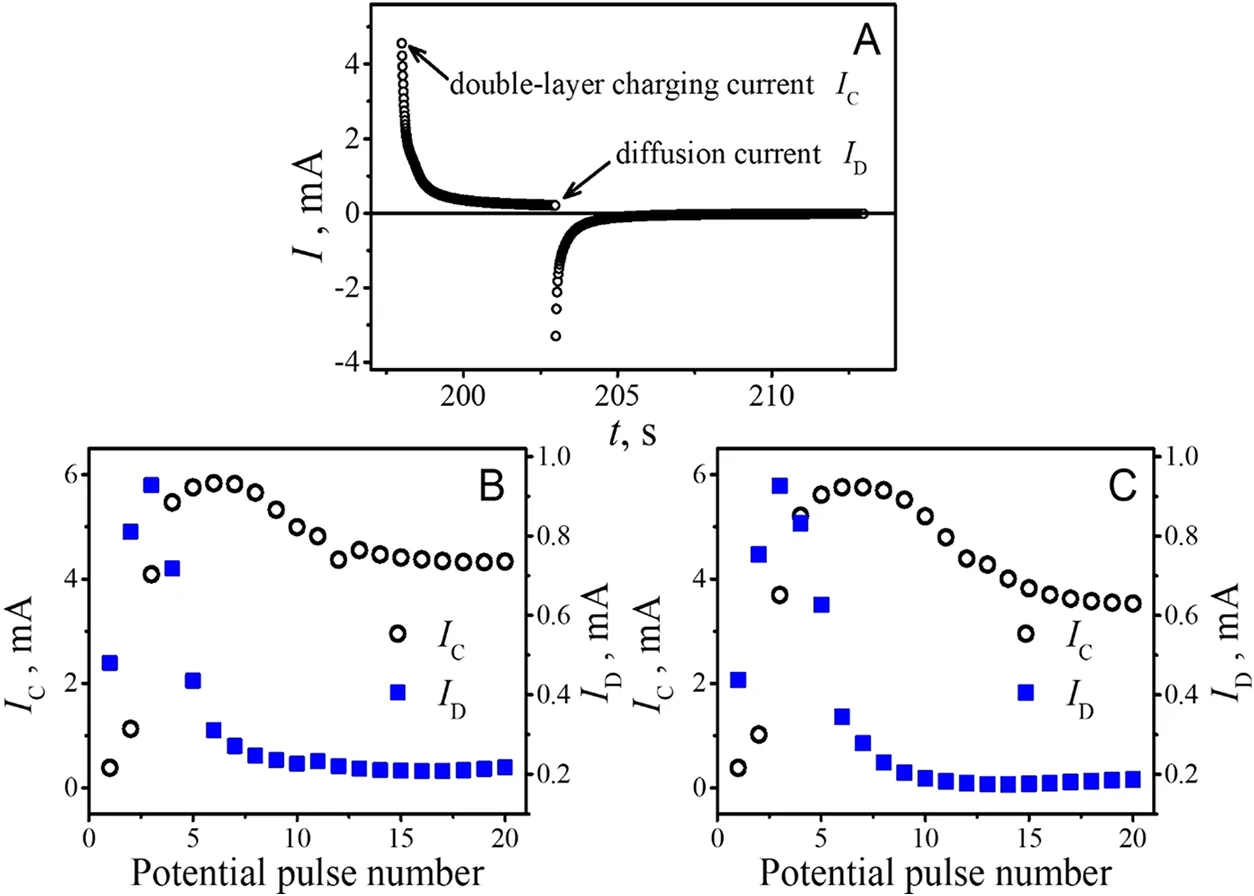
Analysis of the current recorded during the electrochemical deposition of the PPy layer: (A) identification of the analyzed current values. The current at the beginning (open circles) and at the end (closed squares) of electrode reaction during the electrochemical deposition on (B) ITO_(TEMS)_ and (C) ITO_(TMVS)_ electrodes.

The absorbance values at the end of a +1.0 V potential step and at the end of a 0.0 V potential step on the ITO_(TEMS)_ or ITO_(TMVS)_ electrode are depicted in Figure 3B,C. Based on these data, the absorbance shift (Δ*A*) was calculated according to eq 1

1 $$\Delta A=A_{+1.0\;V}-A_{0.0\;V}$$

**Figure 3:**
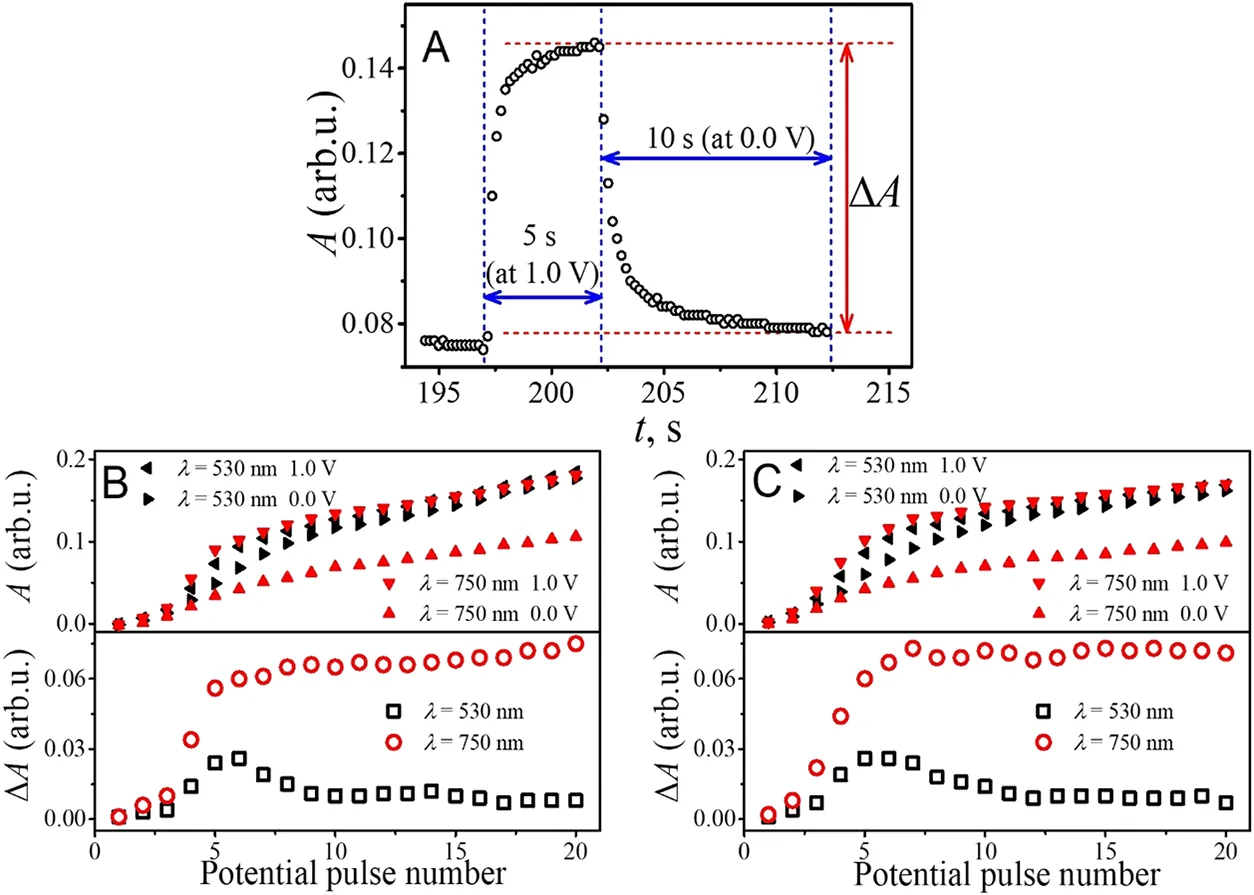
Evaluation of optical absorbance during the electrochemical deposition of the PPy layer: (A) determination of the analytical signal from the analyzed absorbance values with respect to the applied potentials of +1.0 and 0.0 V; the changes of absorbance during the electrochemical deposition of PPy on (B) ITO_(TEMS)_ and (C) ITO_(TMVS)_ electrodes, including the shift of the absorbance observed when the potential was changed from +1.0 to 0.0 V.

The obtained Δ*A* values are shown at the bottom of Figure 3B,C for ITO_(TEMS)_ and ITO_(TMVS)_, respectively. During the DPS-based deposition of PPy, the optical absorbance increased continuously throughout all applied potential pulses, indicating the continuous growth of the PPy layer on the electrode surface. Notably, the Δ*A* values corresponding to the absorbance shift during switching from +1.0 to 0.0 V at 530 nm increased from the first to the sixth potential step, where a maximum was observed, and then decreased during the subsequent steps. In contrast, the Δ*A* values at 750 nm increased more gradually during the same potential-pulsing process and eventually plateaued in the later stages of deposition, without exhibiting a distinct local maximum. We assume that the subtle differences in the characteristics (current, Δ*A*) of Py polymerization on modified electrodes are attributed to the silane functional groups used in this study. As we mentioned above, the methyl and vinyl groups in both silanes are not electrochemically active, and in this system, their role is limited to tuning the hydrophobicity of the ITO surface.

Based on the observed differences in absorbance at 530 and 750 nm, we used the electrochemical polymerization mechanism of pyrrole, as described by Rapta et al.,^34^ to interpret the obtained results. According to this mechanism, when the electrical potential of the PPy layer on the ITO surface changes, the following chemical reactions may occur

2 $${\mathrm{PPy}}^{0}⇄{\mathrm{PPy}}^{•+}+e^{-}$$

3 $${\mathrm{PPy}}^{•+}⇄{\mathrm{PPy}}^{2+}+e^{-}$$

4 $${\mathrm{PPy}}^{0}+{\mathrm{PPy}}^{2+}⇄2{\mathrm{PPy}}^{•+}$$

5 $$2{\mathrm{PPy}}^{•+}\to {{\mathrm{PPy}}_{2}}^{2+}$$

where PPy^0^ is a neutral polymer (oligomer) segment, PPy^•+^ is the corresponding cation radical carrying one positive charge (polaron), and PPy^2+^ is a bication carrying two positive charges (bipolaron). As can be seen, reactions 2 and 3 reflect charge transfer to and from the PPy layer and can therefore be observed electrochemically. Meanwhile, reaction 4 reflects the interaction between two segments as neutral PPy^0^ and bipolaronic PPy^2+^, through the oxidation and reduction processes. However, during this reaction, the total electrical charge of the PPy layer remains unchanged. Therefore, reaction 4 should not significantly affect the electrochemical response. The same can be said for reaction 5, which may reflect the growth of the PPy chain or the cross-linking process, in which two monocharged segments join.

Therefore, when evaluating the data from the chronoamperometric experiment performed using the DPS method, the recorded current (*I*) should be attributed primarily to reactions 2 and 3. At the same time, reactions 4 and 5 are not expected to contribute to the recorded current. However, it should be emphasized that during the electrochemical formation of the PPy layer from the monomer solution, a substantial part of the anodic current is likely associated with oxidation of the pyrrole monomer itself, i.e., with the formation of oligomers and the simultaneous growth of the polymer chain. The overall reaction can be simplified as follows

6 $$n\mathrm{Py}\to {\mathrm{PPy}}_{n}+nH^{+}+ne^{-}$$

In this case, PPy_*n*_ represents a neutral polymer (oligomer) segment. According to the literature, the minimum number of monomer units in the chain, *n*, reported for an oligomer to deposit on the surface is 8.^25^ It should also be noted that the neutral polymer segment PPy_*n*_ shown in reaction 6 corresponds to PPy^0^ presented in reactions 2 and 4. This means that the neutral PPy^0^ segment formed during polymerization can be oxidized in subsequent reaction steps, as shown in reactions 2 and 4.

When the previously discussed reaction model ([Disp-formula eq2]–[Disp-formula eq6]) is evaluated from the perspective of the Vis-spectroscopy experiment, reactions 2 and 4 should increase absorbance in the 530 nm region. In contrast, reactions 3 and 5 should decrease it. This is because reactions 2 and 4 increase the concentration of polarons (PPy^•+^), while reactions 3 and 5 decrease it. At the same time, reactions 3 and 5 increase the concentration of bipolarons (PPy^2+^) in the polymer layer, which should result in increased light absorption in the 750 nm region. Since PPy^2+^ is reduced during reaction 4, the absorbance at 750 nm would be expected to decrease.

From the Vis-spectroscopy data (Figure 4), it can be seen that during the formation of the PPy layer using the DPS method, the overall absorbance increases at both 530 and 750 nm. At first glance, this suggests a continuous increase in the amount of polarons and bipolarons within the forming PPy layer. However, the evaluation of the chronoamperometric data shows that both *I*_C_ and *I*_D_ decrease starting with the seventh pulse (Figure 2). Therefore, to assess the correlation between electrochemical and spectral data, we used the relationship between the chronoamperometrically recorded charge *Q* and the photometrically recorded light absorbance *A*, as commonly applied in spectroelectrochemistry^49–51^

7 $$\Delta A\left(\lambda ,t\right)=\frac{Q\left(t\right)\cdot \varepsilon }{n\cdot F\cdot S}$$

where *F* is the Faraday constant, *S* is the electrode surface area, and *n* indicates the number of electrons transferred across the phase contact surface during the oxidation–reduction reaction. In this equation, Δ*A*(λ*,t*) corresponds to the change in absorbance at wavelength λ after the passage of electric charge *Q* at a specific time *t*. It should be noted that both *A* and *Q* are functions of time *t*, since they are recorded simultaneously during the same chronoamperometric and spectral experiment. In eq 7, ε corresponds to the light absorptivity (or extinction) coefficient at a wavelength λ.

**Figure 4:**
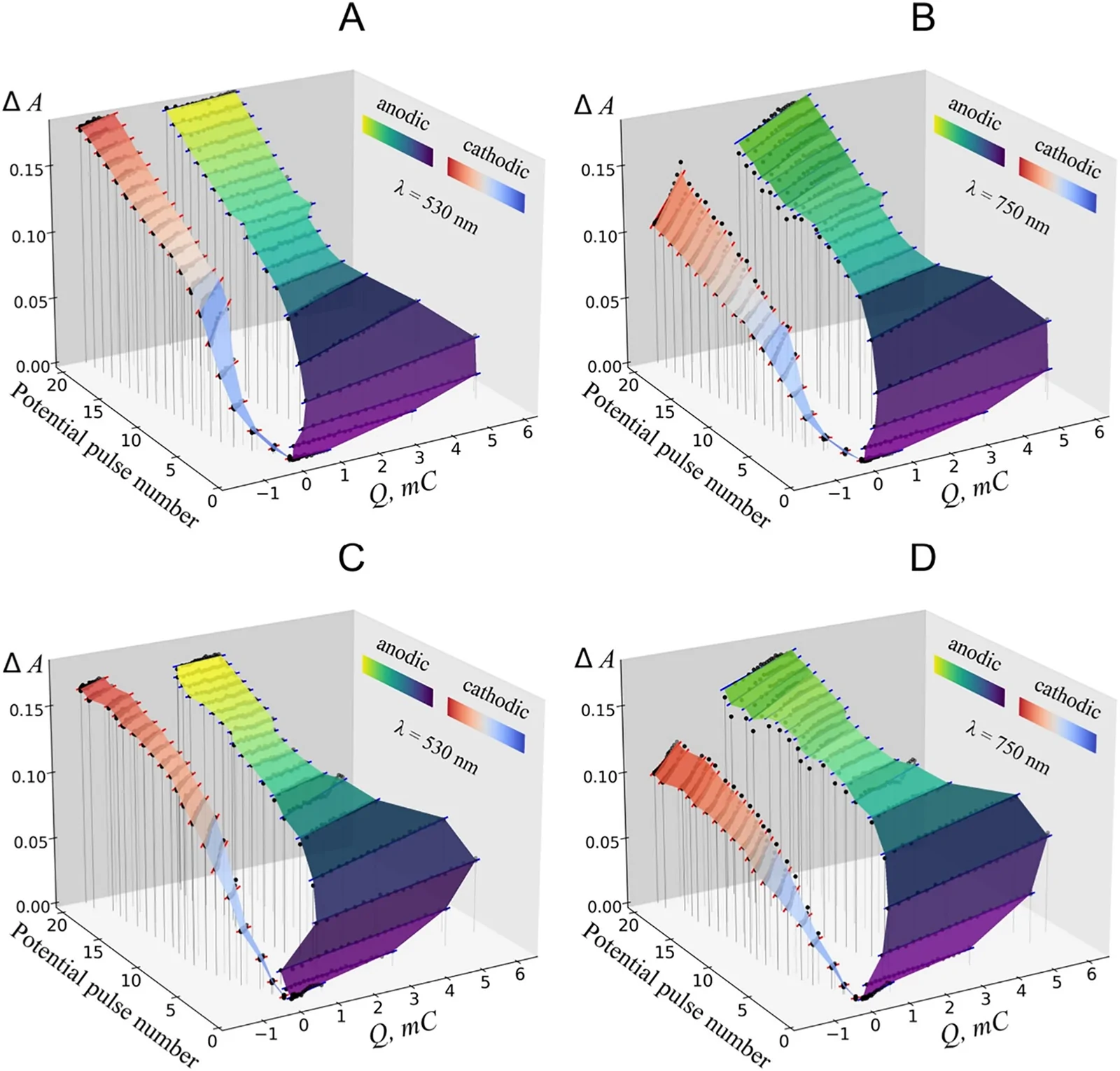
Spectroelectrochemical data showing the dependence of light adsorption on the passed electric charge and pulse number during PPy formation using the DPS method. (A, B) Data obtained with ITO_(TEMS)_ and (C, D) data obtained with ITO_(TMVS)_. The black dots represent the initial data. The straight lines represent the trend lines for the cathodic (red) and anodic (blue) processes. The surfaces represent combined trend lines: a green–purple palette for the anodic process and a blue–red palette for the cathodic process.

In the next stage of data processing, the chronoamperograms obtained during the DPS experiment were integrated over time. This allowed us to obtain the time dependence of the charge *Q*(*t*) passed during each potential pulse. Since Δ*A* and current *I* responses were recorded synchronously, the mutual dependencies of Δ*A* and *Q* were obtained. These dependencies are presented as black dots in the three-dimensional diagrams in Figure 4. Since the DPS method simultaneously recorded the chronoamperograms of the anodic and cathodic pulses, both data series are shown in a single 3D diagram: Figure 4A,B shows the data obtained with ITO_(TEMS)_ at wavelengths 530 and 750 nm, respectively, and Figure 4C,D shows the data obtained with ITO_(TMVS)_.

By applying the spectroelectrochemical linear relationship given in eq 7, i.e., Δ*A* = *f* (*Q*), the experimental data were fitted. This resulted in lines shown in red (cathodic) and blue (anodic) in Figure 4. By connecting these lines, three-dimensional surfaces were obtained, representing the anodic Δ*A* = *f* (*Q*) variation for each pulse (yellow–purple color palette) and the cathodic variation (red–blue color palette). Several characteristic trends can be observed from the resulting three-dimensional surfaces. First, in all the changes shown in Figure 4, the anodic charge during the DPS experiment was greater than the cathodic charge. This reflects the irreversible oxidation of pyrrole according to reaction 6, together with the oxidation of PPy according to reactions 2 and 3. It can also be seen that the charge increased significantly up to the third anodic potential pulse and then began to decrease from the fourth pulse onward. In contrast, no comparable trends were observed for the cathodic process, where the charge increased only slightly with each pulse.

By comparing the Δ*A* = *f*(*Q*) dependencies, it can be seen that at 750 nm, the absorbance Δ*A* depends more strongly on the passed charge *Q* than at 530 nm. This effect is more pronounced in the cathodic process, as shown by the red–blue surfaces in Figure 4B,D. Meanwhile, in the anodic process, the influence of charge *Q* on Δ*A* becomes more pronounced after ∼10 pulses. Therefore, this nonidentical relationship between Δ*A* and *Q* at different wavelengths prompted us to examine the obtained data in greater depth. Since eq 7 reflects the linear dependence of Δ*A* on *Q*, the “slope” of this relationship was evaluated for the anodic *k*_an_ (eq 8) and cathodic *k*_cat_ (eq 9) potential pulses

8 $$k_{\mathrm{an}}=\frac{\varepsilon }{n_{\mathrm{ox}}\cdot F\cdot S}$$

and

9 $$k_{\mathrm{cat}}=\frac{\varepsilon }{n_{\mathrm{red}}\cdot F\cdot S}$$

The variables may include the absorptivity (extinction) coefficient *ε* and the value *n*, which denotes the number of electrons transferred across the phase boundary during oxidation (*n*_ox_) or reduction (*n*_red_). We assumed that ε remains constant for the corresponding cathode and anode pulses (e.g., the first, second, etc.). Under this assumption, dividing the slope of the anodic pulse *k*_an_ by that of the cathodic pulse *k*_cat_ gives the electron number ratio *n*_ox_/*n*_red_. This value reflects the ratio of electrons participating in the oxidation process (*n*_ox_) to those participating in the reduction process (*n*_red_). The corresponding results are shown in Figure 5.

**Figure 5:**
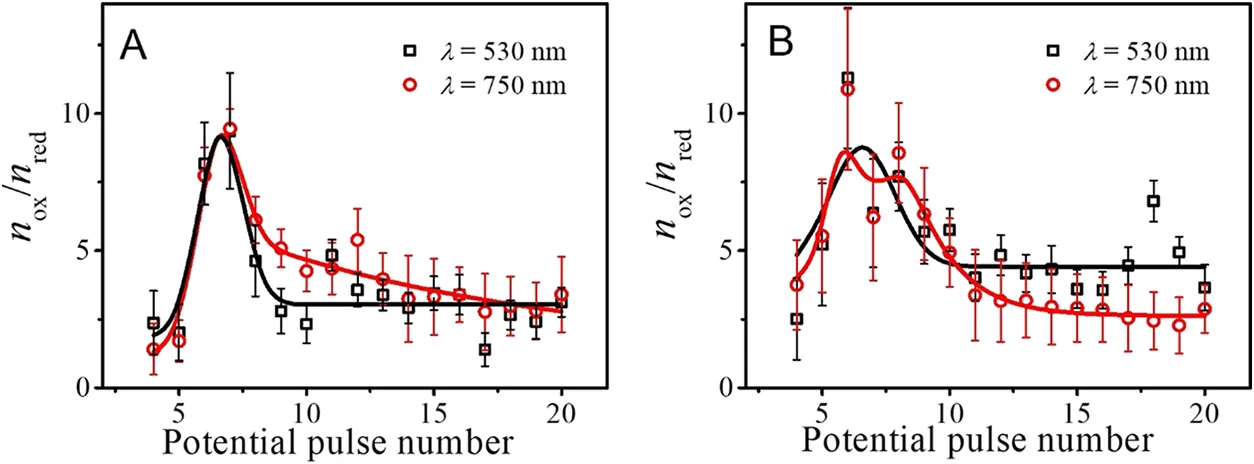
Dependence of the electrical charge ratio *n*_ox_/*n*_red_ for the oxidation and reduction processes on the potential pulse during PPy layer formation by the DSP method. The black dots and the middle line correspond to data recorded at 530 nm, whereas the red dots and the line correspond to data measured at λ = 750 nm. (A) Data for ITO_(TEMS)_. (B) Corresponding data for ITO_(TMVS)_.

First, the *n*_ox_/*n*_red_ ratio is greater than 1 across the entire potential pulse range. This indicates that, throughout the DPS experiment, the anodic process involved a higher net charge transfer than the cathodic process. Based on the ratio values, the average charge associated with the anodic pulses was approximately five times higher than that associated with the cathodic pulses. In this case, *n*_ox_ reflects the electrons (charge) involved during the anodic step of reactions 2, 3, and 6. Meanwhile, *n*_red_ reflects the electrons involved during the cathodic step in the reverse reactions of [Disp-formula eq2] and 3. It can also be observed that the *n*_ox_/*n*_red_ ratio is not constant throughout the pulse sequence, indicating that the formation of the PPy layer did not proceed at a uniform rate. The highest *n*_ox_/*n*_red_ values for both ITO_(TEMS)_ (Figure 5A) and ITO_(TMVS)_ (Figure 5B) were observed at potential pulses 5–6. Comparing the *n*_ox_/*n*_red_ data recorded at 530 nm (black squares) with those measured at 750 nm (red circles), it can be seen that at 530 nm, the ratio became nearly constant after 5–6 potential pulses, reaching values of approximately 3 for ITO_(TEMS)_ and 4 for ITO_(TMVS)_. Meanwhile, at 750 nm, the *n*_ox_/*n*_red_ ratio gradually decreased from its maximum value (at 5–6 pulses) until the end of the experiment.

As mentioned earlier, at 530 nm, light absorbance is more closely related to the presence of polarons, i.e., PPy^•+^ in the polymer layer. In contrast, at 750 nm, absorbance is more sensitive to the concentration of bipolarons PPy^2+^. Therefore, several preliminary conclusions can be drawn from the *n*_ox_/*n*_red_ trends shown in Figure 5. First, during electrochemical formation of the PPy layer on the silanized ITO surface, the resulting polymer is predominantly in an oxidized state, i.e., in the form of polarons and/or bipolarons. This observation is consistent with that reported by Rapta et al.^34^ It should also be noted that at 750 nm, the *n*_ox_/*n*_red_ ratio shows a decreasing tendency after pulses 5–6, indicating that the concentration of bipolarons PPy^2+^ in the growing layer also decreases. This is likely related to slowing of the forward reactions 3 and 5 and/or the acceleration of reaction 4.

Figure 5 shows ITO_(TEMS)_ and ITO_(TMVS)_, after 10 pulses: for TEMS, a roughly coincident *n*_ox_/*n*_red_ trend is observed at both 530 and 750 nm. Meanwhile, for TMVS, the 530 nm *n*_ox_/*n*_red_ values (Figure 5B, black dots) are slightly higher than those at 750 nm (Figure 5B, red dots). This indicates a higher concentration of polarons rather than that of bipolarons. Both TEMS and TMVS silanes form an interfacial barrier on the ITO surface but do not participate in the PPy polymerization. Because their side functional groups are structurally similar and lack covalent bonding with the polymer, there is a similar dependence trend of the electrical charge ratio *n*_ox_/*n*_red_ for the oxidation and reduction processes.

Overall, the obtained results provide insights into the key processes governing DPS-enabled electropolymerization of PPy on the silane-modified ITO electrodes. The combined chronoamperometric and *in situ* Vis-spectroscopy measurements show that the polymer formation does not proceed at a constant rate but instead begins with initial growth, followed by a phase of accelerated growth, and then transitions to more stable behavior. The *n*_ox_/*n*_red_ ratio remaining above 1 throughout the pulse sequence indicates that the anodic step involves a higher net charge transfer than the cathodic step. This behavior is consistent with contributions not only from reversible oxidation of the growing PPy layer but also from irreversible pyrrole oxidation and chain growth processes, resulting in net layer growth during successive pulses. The highest *n*_ox_/*n*_red_ values, observed around pulses 5–6, suggest an intermediate stage in which polymerization is most active, followed by a gradual transition to slower and more stable growth. At the same time, the wavelength-dependent absorbance changes indicate that different charge carrier states are formed during deposition: the maximum in the 530 nm response observed around pulses 5–6 is associated with changes in the amount of polarons, whereas the gradual increase and plateau-like behavior at 750 nm suggest progressive formation and subsequent stabilization of bipolaron states, respectively. Together, these findings show that PPy electropolymerization under pulsed conditions results from the combined effects of oxidation–reduction processes, net polymer formation, changes associated with the polaron and bipolaron states, and substrate-dependent interfacial interactions. At the same time, *in situ* Vis spectroscopy data proved to be an effective approach for tracking these processes during deposition, providing deeper insights into the PPy layer formation.

To demonstrate the applicability of ITO_(TEMS)_/PPy and ITO_(TMVS)_/PPy electrodes alongside the main test spectroelectrochemical assessment of PPy formation dynamics of this study, the prepared ITO_(TEMS)_/PPy and ITO_(TMVS)_/PPy electrodes were further evaluated as the optical pH-sensitive layers in Britton–Robinson buffer (BRB) solutions over an approximate pH range of 2 to 10 (Figure 6). Upon changing the solution pH, the corresponding changes in Vis spectra were analyzed, focusing on the absorbance responses at 530 and 750 nm. Figure 6A,C shows that the Vis absorbance spectra of both ITO_(TEMS)_/PPy and ITO_(TMVS)_/PPy layers vary with solution pH, confirming that the deposited PPy layers exhibit pH-dependent optical behavior. As shown in Figure 6B,D, both electrodes exhibited pronounced pH-dependent optical response at 530 nm. For the ITO_(TEMS)_/PPy, sensitivities of –4.55 × 10^–3^ arb. u. pH^–1^ at 530 nm over the pH interval of 2.3–7.3 and 4.34 × 10^–3^ arb. u. pH^–1^ at 750 nm over the pH range 7.3–10.4 were obtained from the selected linear regions. In comparison, ITO_(TVMS)_/PPy exhibited sensitivities of −3.82 × 10^–3^ arb. u. pH^–1^ at 530 nm over the pH range of 2.4–9.0 and 3.58 × 10^–3^ arb. u. pH^–1^ at 750 nm over the pH interval of 6.1–10.8. Notably, the ITO_(TMVS)_/PPy electrode exhibited linear responses over broader pH intervals at both wavelengths compared with the ITO_(TEMS)_/PPy electrodes. The corresponding changes in optical absorbance with variation in pH are expected due to protonation and deprotonation processes within the oxidized PPy film, which may lead to redistribution of π-electrons along the conjugated polymer backbone and local structural changes between benzenoid- and quinoid-like forms of PPy. Since electropolymerized PPy exists in a partially oxidized (doped) state, these processes may further influence the local equilibrium and relative contribution of neutral, polaronic, and bipolaronic forms within the polymer matrix. Such changes in the electronic structure and charge delocalization may result in the modification of optical absorbance properties of the polymer layer.^19,35,37,39,47,52–56^

**Figure 6:**
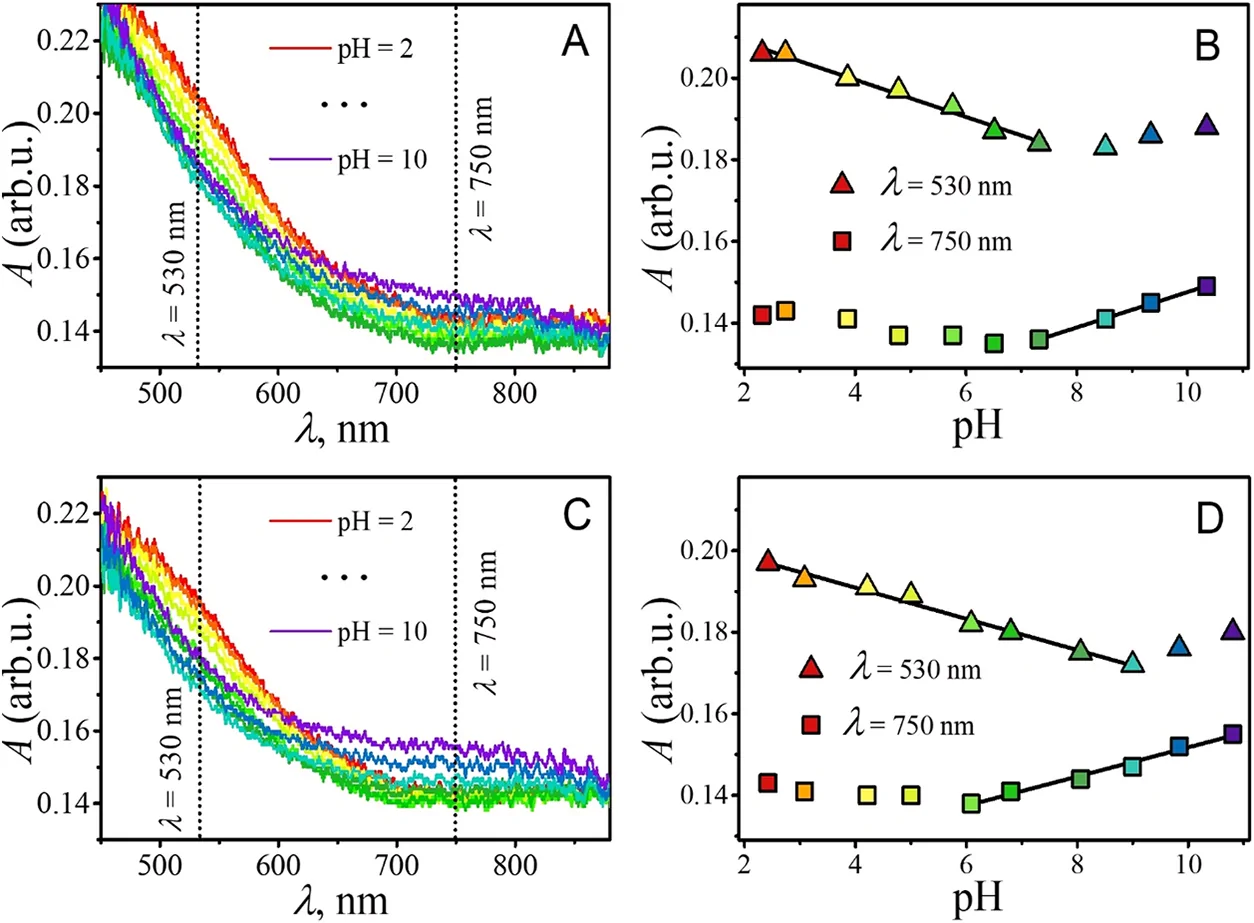
Vis light absorbance spectra of PPy layers at different pH values after electrochemical deposition on (A) ITO_(TEMS)_ and (C) ITO_(TMVS)_ electrodes. Dependence of the optical absorbance of PPy layers on solution pH at 530 nm and at 750 nm for (B) ITO_(TEMS)_ and (D) ITO_(TMVS)_ electrodes.

Overall, these results indicate that the signal at 530 nm is more suitable for pH sensing in the acidic to neutral region, whereas the signal at 750 nm may be more useful in the alkaline region. In addition, the secondary response at 750 nm could help distinguish ambiguous absorbance values obtained at λ = 530 nm, thereby indicating whether the sample pH lies within the primary functional range or outside it. Among the two investigated systems, ITO_(TMVS)_/PPy exhibits a slightly broader usable pH range and marginally improved sensing performance. This behavior may be related to differences in layer formation during electropolymerization. As discussed above, the TMVS interfacial layer may partially hinder charge transfer between the ITO substrate and the growing PPy layer during deposition. In addition, after approximately 5–6 potential pulses, the *n*_ox_/*n*_red_ ratio at λ = 530 nm reached stable values of about 3 for ITO_(TEMS)_ (Figure 5A) and about 4 for ITO_(TMVS)_ (Figure 5B). The slightly higher ratio observed for the TMVS-modified electrode may suggest that the PPy layer formed under somewhat more oxidizing conditions, with slight differences in the relative contribution of polarons, bipolarons, and quinoid-like forms of oxidized PPy compared with the TEMS-modified electrode. These differences during deposition may lead to minor changes in the structure of the resulting PPy layer. This may explain the broader pH response observed for the ITO_(TMVS)_/PPy electrode.

It should also be noted that, in the present work, surface modification of the ITO electrodes with TEMS and TMVS was employed to improve the adhesion and mechanical stability of the deposited PPy layers, as PPy formed on the untreated ITO substrates is prone to partial delamination or surface damage during repeated solution exposure. In the present study, both ITO_(TEMS)_/PPy and ITO_(TMVS)_/PPy electrodes remained visually intact throughout the pH-sensing experiments with no observable peeling or mechanical degradation after immersion in BRB solutions at different pH values. These results indicate that silanization provided effective interfacial anchoring of the PPy layer under the tested conditions. Maintaining layer integrity is important for practical optical sensing applications, where reproducible responses depend on maintaining a stable polymer layer during multiple measurements.

## Conclusions

Polypyrrole layers were electrodeposited on the TEMS- and TMVS-modified ITO electrodes using the DPS method. Combined chronoamperometric and *in situ* Vis light absorption spectroscopy measurements showed that the formation of the conducting polymer did not proceed at a constant rate but involved an initial growth stage, followed by an accelerated polymerization around pulses 5–6, and a subsequent transition to more stable layer growth. The *n*_ox_/*n*_red_ ratio remained greater than 1 throughout the deposition process, indicating that anodic charge transfer exceeded the cathodic charge transfer because of simultaneous polymer oxidation and net pyrrole electropolymerization. The optical response monitored at 530 and 750 nm indicated changes in the relative amounts of polaronic and bipolaronic species during the deposition. The higher *n*_ox_/*n*_red_ values observed for the TMVS-modified electrode at 530 nm suggest that the corresponding PPy layer was formed under more oxidizing conditions, with a relatively higher fraction of polaronic species compared with the TEMS-modified ITO. These results further demonstrate that electrochromic monitoring can be employed not only for practical electrochromic applications but also as an effective tool for *in situ* study of polymerization behavior and gaining deeper insights into the formation and electronic properties of the conducting polymer layer. The obtained PPy layers also exhibited pH-dependent optical behavior in the buffer solutions. Among the tested electrodes, ITO_(TMVS)_/PPy showed the broader functional pH sensing range, while the response at λ = 530 nm was more suitable in the acidic to neutral region, and the response at 750 nm was more useful under alkaline conditions. In addition, both silane-modified electrodes maintained good layer integrity during pH measurements, confirming that surface silanization improved adhesion and mechanical stability of the deposited PPy layers.

## Materials and Methods

### Chemicals and Instrumentation

Ultrapure water with a conductivity of 0.055 μS cm^–1^ was purified using the Adrona Crystal 7 water purification system and used to prepare all solutions. The pyrrole was purchased from Sigma-Aldrich (Steinheim, Germany) and distilled before use. All other reagents were of “analytical grade” and were used as obtained. Chemical reagents such as boric acid from Scharlau (Sentmenat, Spain), acetic acid from Carl Roth, (Karlsruhe, Germany), phosphoric acid from Fluka (Buchs, Germany), lithium perchlorate from Alfa Aesar (Kandel, Germany), sodium hydroxide and trimethoxyvinylsilane (TMVS) from Merck (Darmstadt, Germany), triethoxymethylsilane (TEMS) from Sigma-Aldrich (Steinheim, Germany), ammonium hydroxide and hydrogen peroxide from Chempur (Piekary Ślaskie, Poland), and acetone from Reachem (Bratislava, Slovakia) were used.

A USB4000-FL spectrometer equipped with SpectraSuite software from Ocean Optics (Largo, USA) was used for all optical measurements. Two different glass cuvettes were used for electrochemical deposition of PPy and electrochemical/optical investigations: (i) a glass cuvette (with dimensions of *H* × *W* × *D* = 32 mm × 30 mm × 18 mm) was used for the electrochemical formation of the PPy layer and (ii) another, wider glass cuvette (with dimensions of *H* × *W* × *D* = 32 mm × 50 mm × 18 mm) was used for the electrochemical/optical evaluation of formed ITO/PPy properties.

A computer-controlled potentiostat/galvanostat PGSTAT 128N, equipped with Nova 1.10 software from Eco-Chemie (Utrecht, The Netherlands), was used for electrochemical deposition of conducting polymer-based layers and for electrochemical measurements. A three-electrode system was applied for all electrochemical experiments. An indium tin oxide (ITO)-coated glass slide was purchased from Sigma-Aldrich (Steinheim, Germany). The ITO with a surface resistivity of 15–25 Ω cm^–2^ was used as a working electrode. An Ag/AgCl_(3 M KCl)_ electrode from CH Instruments (Austin, USA) was used as the reference electrode, and a platinum wire was used as the counter electrode.

### Pretreatment of the ITO Electrode and the Silanization Procedure

Before polymer deposition, all ITO electrodes underwent a two-step pretreatment: (1) ITO surface cleaning and activation and (2) silanization (Figure 7, first and second steps).

**Figure 7:**
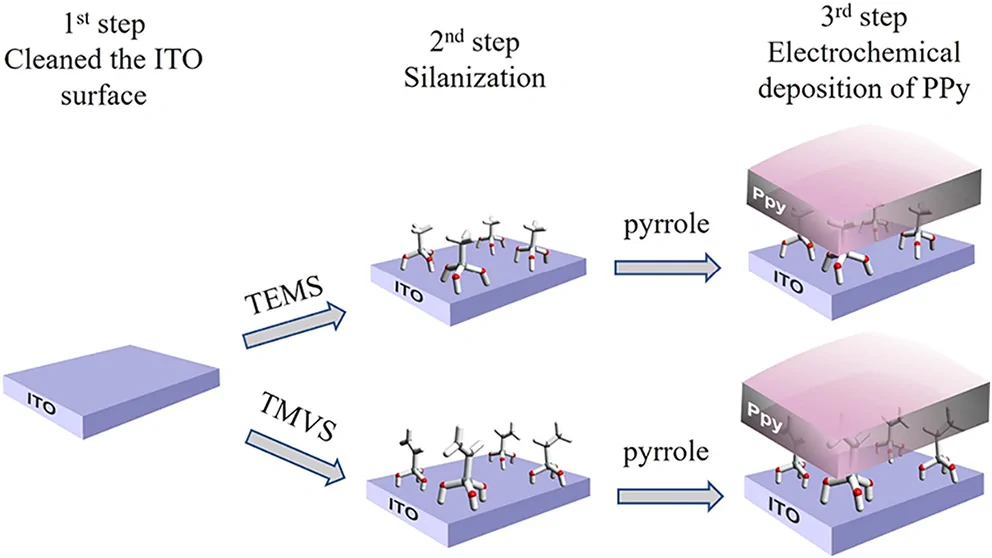
Schematic of PPy layer formation on the ITO surface: Step 1: silanization with TEMS or TMVS and Step 2: PPy layer formation using the DPS method.

The first pretreatment step and modification of the ITO electrodes were carried out according to the procedure described in our previous study.^16^ The ITO electrode surface was washed for 5 min in a 27% NH_4_OH and 30% H_2_O_2_ solution at 3:1 (v/v) ratio and preheated to 50 °C. After that, the ITO electrodes were sequentially cleaned in an ultrasonic bath in water, acetone, and water again for 15 min in each liquid.

The second pretreatment step: the ITO surface was modified with silane by immersing the cleaned electrodes overnight in a 4% (v/v) TEMS or TMVS solution in acetone. As a result, TEMS- or TMVS-modified surfaces (ITO_(TEMS)_ or ITO_(TMVS)_) were formed. The excess silane was removed by rinsing the electrodes with acetone and then ultrapure water. ITO_(TEMS)_ and ITO_(TMVS)_ were then dried under an argon stream and stored under dry conditions until further use.

### Electrochemical Deposition of a Polypyrrole Layer on ITO_(TEMS)_ and ITO_(TMVS)_ Electrodes (Figure 7, Third Pretreatment Step)

The electrochemical deposition of PPy layers on ITO_(TEMS)_ and ITO_(TMVS)_ electrodes was carried out at room temperature in an aqueous solution containing 10 mM pyrrole and 0.1 M LiClO_4_ as the electrolyte. Pyrrole was electropolymerized on the modified ITO surfaces by applying a DPS protocol of +1.0 V for 5 s and then by 0 V vs Ag/AgCl_(3 M KCl)_ for 10 s, for a total of 20 pulses at each potential, resulting in the corresponding ITO_(TEMS)_/PPy and ITO_(TMVS)_/PPy layers. During polymerization, *in situ* light absorption spectroscopy measurements were performed, and absorbance changes were monitored at 530 and 750 nm for comparative analysis.

### Evaluation of ITO_(TEMS)_/PPy and ITO_(TMVS)_/PPy Electrodes for Optical pH Sensing

Britton–Robinson buffer (BRB) solution was prepared, and it comprised 0.01 M boric acid, 0.01 M acetic acid, and 0.01 M phosphoric acid.^57^ The ionic strength of BRB was supported with 0.1 M LiClO_4_. The pH of the buffer was adjusted to the selected values by adding 1 M NaOH and monitored with a ProLine Plus pH meter (Q-i-s, China).

The optical pH sensing performance of the ITO_(TEMS)_/PPy and ITO_(TVMS)_/PPy electrodes was evaluated in BRB solutions of different pH values. Vis light absorption spectra were recorded after equilibration at each pH. For comparative analysis, absorbance changes were monitored at 530 and 750 nm.

## Abbreviations

PPy: polypyrrole
ITO: indium tin oxide
FTO: fluorine-doped tin oxide
DPS: double potential step
TEMS: triethoxymethylsilane
TMVS: trimethoxyvinylsilane
CV: cyclic voltammetry
UV–Vis: ultraviolet–visible spectroscopy
Vis: visible light
BRB: Britton–Robinson buffer
ITO_(TEMS)_: TEMS-modified indium tin oxide electrode
ITO_(TMVS)_: TMVS-modified indium tin oxide electrode
